# Dynamic skeletal muscle loss and its predictive role on 90-day mortality in patients with acute-on-chronic liver failure

**DOI:** 10.3389/fnut.2025.1446265

**Published:** 2025-02-27

**Authors:** Nan Geng, Ming Kong, Jiateng Zhang, Manman Xu, Huina Chen, Wenyan Song, Yu Chen, Zhongping Duan

**Affiliations:** ^1^Department of Infectious Diseases, Nanjing Drum Tower Hospital, Affiliated Hospital of Medical School, Nanjing University, Nanjing, China; ^2^Fourth Department of Liver Disease, Beijing Youan Hospital, Capital Medical University, Beijing Municipal Key Laboratory of Liver Failure and Artificial Liver Treatment Research, Beijing, China; ^3^Beijing Tiantan Hospital, Capital Medical University, Beijing, China; ^4^Department of Clinical Epidemiology and Clinical Trial, Capital Medical University, Beijing, China; ^5^Department of Radiology, Beijing Youan Hospital, Capital Medical University, Beijing, China

**Keywords:** acute-on-chronic liver failure, skeletal muscle loss, sarcopenia, malnutrition, short-term outcome

## Abstract

**Background:**

Low skeletal muscle mass is an independent risk factor for increased mortality in patients with acute-on-chronic liver failure (ACLF). However, no study has evaluated the temporal changes in muscle mass during the course of ACLF. Therefore, this study aimed to investigate the dynamic changes in muscle mass and their prognostic role in patients with ACLF.

**Methods:**

A retrospective analysis was conducted on consecutive patients with ACLF who underwent two or more abdominal computed tomography examinations within 90 days of admission. The percentage change rates of the skeletal muscle index at the third lumbar vertebra (L3-SMI) were calculated as (L3-SMI_final_ - L3-SMI_initial_)/(L3-SMI_initial_) × 100%.

**Results:**

A total of 154 patients with ACLF were included. During the course of ACLF, the percentage change rates of L3-SMI at 2–7, 8–14, 15–30, 31–60, and 61–90 days were − 0.83 ± 4.43, −3.76 ± 4.40, −7.30 ± 5.89, −10.10 ± 7.45, and − 5.53 ± 9.26, respectively. Significant reductions in L3-SMI were noted in patients with severe conditions compared to other patients at 2–7 days and 15–30 days. Moreover, the rate of decrease in L3-SMI in patients with a lower respiratory quotient (RQ) was significantly greater than that in patients with a normal RQ at 2–7 days and 15–30 days. Additionally, high muscle loss (HR 2.059; 95% CI 1.122–3.780, *p* = 0.020), rather than pre-existing sarcopenia (HR 1.430; 95% CI 0.724–2.826, *p* = 0.303) at baseline, was independently associated with 90-day mortality.

**Conclusion:**

Deterioration in muscle mass is associated with disease severity and poor nutritional status and serves as a more effective predictor of adverse short-term outcomes in patients with ACLF. These findings underscore the importance of dynamic evaluation of muscle loss and emphasize the necessity of reversing muscle loss in patients with ACLF.

## Graphical abstract

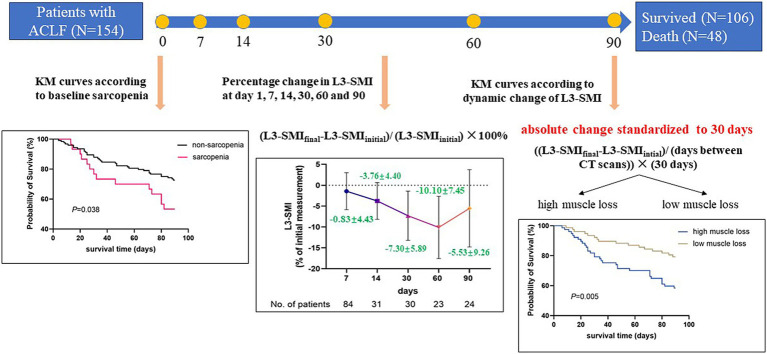

## 1 Introduction

Acute-on-chronic liver failure (ACLF) is a clinical syndrome characterized by acute decompensation of liver function in patients with chronic liver disease, accompanied by multiple organ failure and high short-term mortality (1). Low skeletal muscle mass (sarcopenia) is an important prognostic indicator in patients with ACLF (2–5). Under these clinical conditions, skeletal muscle mass is measured simultaneously. However, compared to chronic diseases in which skeletal muscle depletion occurs more slowly, ACLF may significantly increase muscle proteolysis and decrease muscle quantity owing to systemic inflammation, metabolic abnormalities, bed rest, and reduced dietary intake (6).

Previous studies conducted on critically ill patients have shown that severe muscle loss is independently associated with higher morbidity and mortality (7, 8). Because the muscle mass of patients with severe acute disease undergoes significant changes over time, the change in muscle mass may serve as a more effective predictor of clinical outcomes than the initial muscle mass at baseline. Although several studies have explored the incidence and prognostic role of low muscle mass in patients with ACLF, no study has investigated the impact of ACLF on temporal changes in skeletal muscle mass and adverse outcomes associated with different degrees of muscle loss.

Cross-sectional image analysis of computed tomography (CT) scans of the abdomen, especially the third lumbar vertebra, has become a precise and reproducible approach for quantifying muscle mass (9). Although protocols for CT scans with definite intervals in the management of patients with ACLF are lacking, the analysis of temporally distinct scans using standardized time intervals has been used to determine the rate of muscle loss (10). The objectives of the present study were to (1) identify the dynamic changes in skeletal muscle mass during the course of ACLF, (2) investigate the factors that affect the rate of skeletal muscle mass loss, and (3) determine whether a change in muscle mass is an independent risk factor for 90-day mortality in patients with ACLF.

## 2 Materials and methods

### 2.1 Ethics approval

This retrospective study conformed to the Declaration of Helsinki and was approved by the Ethics Committee of Beijing Youan Hospital, Beijing, China. The requirement for informed consent was waived because of the study’s retrospective nature and because the patients’ privacy was not compromised.

### 2.2 Study design and participants

A retrospective analysis was conducted on consecutive hospitalized patients with ACLF between January 2019 and August 2022 at Beijing Youan Hospital, Capital Medical University (Beijing, China). The inclusion criteria were as follows: (1) diagnosis of ACLF based on the Asian Pacific Association for the Study of the Liver (APASL) consensus, (2) age > 20 years. The exclusion criteria were as follows: (1) presence of liver cancer or other malignant tumors, (2) severe extrahepatic organ diseases, (3) complicated with consumptive diseases (4) neuromuscular diseases or long-term bedridden status, (5) long-term glucocorticoid therapy, (6) unavailable height or weight data, (7) loss to follow-up, (8) without available abdominal CT scans 2 weeks following admission or absence of 2 sequential CT images within 90 days, and (9) liver transplantation within 90 days.

### 2.3 Clinical parameters

Clinical data, including age, sex, height, weight, pre-existing liver cirrhosis, manifestations at admission (e.g., ascites, hepatic encephalopathy (HE), acute kidney injury (AKI), and infection), and baseline laboratory parameters (e.g., total bilirubin, albumin, creatinine, sodium, lactic acid, international normalized ratio (INR), hemoglobin, white blood cell (WBC) count, and platelet count), were retrieved from electronic medical records. Liver disease severity was assessed using the Model for End-stage Liver Disease-Sodium (MELD-Na) and APASL ACLF Research Consortium (AARC) scores. Additionally, according to the European Association for the Study of the Liver-Chronic Liver Failure Consortium (EASL-CLIF-C) diagnostic criteria, we further identified the CILF-C ACLF and no CLIF-C ACLF groups.

Body mass index (BMI) was calculated as dry weight (kg)/height squared (m^2^), and ≥ 25 kg/m^2^ was defined as overweight. Dry weight was measured considering fluid retention by subtracting a certain body weight (mild, 5%; moderate, 10%; severe, 15%; and 5% in peripheral edema) (11). Patients were followed up using a medical record system or telephone. The outcome was death within 90 days of listing.

### 2.4 Subjective global assessment, anthropometric and frailty measurements

SGA is evaluated based on weight loss, dietary intake, gastrointestinal symptoms, changes in daily activities, disease stress, subcutaneous fat, upper arm muscle circumference, and peripheral edema. Therefore, patients were categorized into three groups: A (good nutrition), B (moderate malnutrition), and C (severe malnutrition) (12). Mid-arm circumference (MAC) and triceps skinfold thickness (TSF) were evaluated using a tape measure and skinfold thickness gage. The mid-arm muscle circumference (MAMC) was calculated using the following formula: MAMC = MAC (cm) - *π* × TSF (cm). Each patient underwent three measurements to obtain the average value. The liver frailty index (LFI) was evaluated using three performance-based tests (grip strength, chair stand, and balance) and was calculated using the formula: LFI = (−0.33 × gender-adjusted grip strength) + (−2.529 × number of chair stands per second) + (−0.04 × balance time) + 6. Frailty was defined as LFI ≥ 4.5 (13).

### 2.5 Respiratory quotient evaluation

The RQ was measured using a cardiorespiratory diagnostic system for nutritional metabolism (Medgraphics Corporation, Saint Paul, MN, United States). The test was conducted in the morning within a quiet and moderately temperature-controlled room (24–26°C). Each patient had 30 min of bed rest and fasted for at least 2 h. The gas analyzer and volume measurement equipment were calibrated before performing the test. RQ was calculated as VCO_2_/VO_2,_ and RQ < 0.8 was defined as low RQ (14).

### 2.6 CT scan analysis

Quantitative assessments of skeletal muscle mass (SMA, cm^2^) at the third lumbar vertebra (L3) on abdominal scans were performed using the image analysis software SliceOmatic (V5.0; Tomovision, Magog, Canada). A Hounsfield unit (HU) range of −29 to 150 was used to measure skeletal muscle (SM). The cross-section of the L3-SMA was normalized for height squared to obtain the L3-skeletal muscle index (SMI, cm^2^/m^2^) (15). Using our established protocol, we defined sarcopenia as ≤40.2 cm^2^/m^2^ in men and ≤ 31.6 cm^2^/m^2^ in women (16). As previously reported, the percentage change in L3-SMI was expressed as (L3-SMI_final_ - L3-SMI_initial_) / (L3-SMI_initial_) × 100%, and the absolute change standardized to 30 days was expressed as ((L3-SMI_final_ - L3-SMI_intial_) / (days between CT scans)) × (30 days) (10). The absolute change standardized to 30 days was dichotomized according to the median value, and the patients were stratified into high and low muscle loss groups (17).

### 2.7 Statistical analysis

Kolmogorov–Smirnov (n ≥ 50) and Shapiro-Wilks (n < 50) tests were utilized to assess normal distribution in our study. Normally and non-normally distributed quantitative variables are presented as the mean ± SD and median with interquartile range and were compared using Student’s t-test and the Wilcoxon-Mann test, respectively. Qualitative variables are presented as numbers (%) and were compared using the chi-square test. Cumulative survival curves were generated using the Kaplan–Meier method and compared using the log-rank test. The relationship between skeletal muscle loss and survival probability was analyzed using univariate and multivariate Cox proportional hazards models. We excluded HE, total bilirubin, creatinine, INR, sodium, and serum lactic acid levels from the multivariate Cox regression analysis to avoid collinearity because these variables were included in the MELD-Na and AARC scores. Time-dependent receiver operating characteristics (ROC) curves, and the corresponding area under the curve (AUC) values were used to compare the predictive performance of sarcopenia, high muscle loss, MELD-Na, and AARC for 90-day mortality. A *p*-value of less than 0.05 was considered statistically significant. Statistical analyses were performed using SPSS (version 24.0, IBM SPSS, Chicago, IL, United States) and R × 64 4.3.2.

## 3 Results

### 3.1 Study population and baseline characteristics

**Figure 1:**
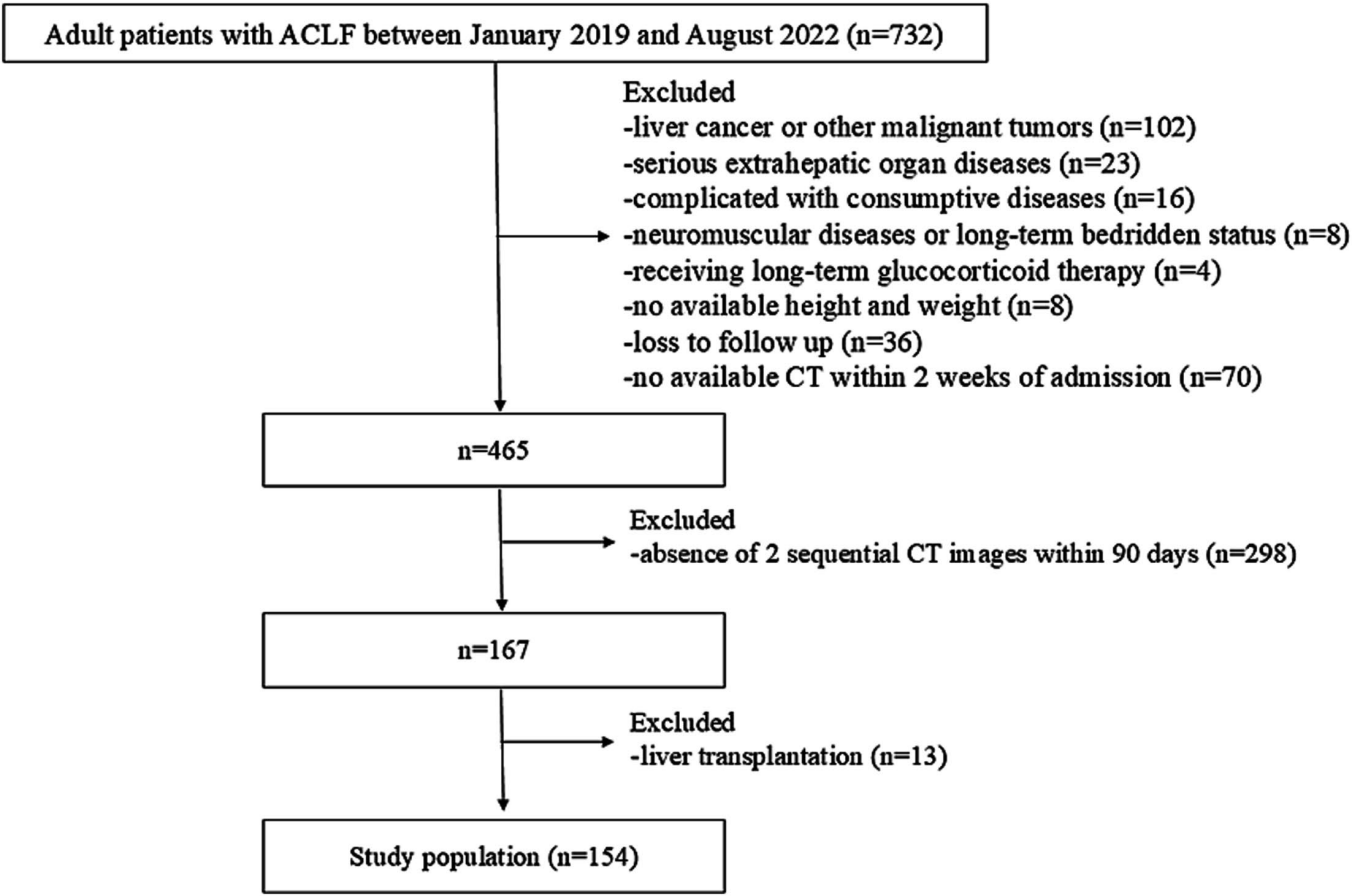
Flow diagram for enrolment in the study.

Among 732 consecutive adult patients diagnosed with ACLF beween January 2019 and August 2022, a total of 154 patients were enrolled in the study (Figure 1). Of the 154 patients who underwent at least two CT scans within 90 days of hospitalization, 124 had 2 CT scans, 23 had 3, 6 had 4, and 1 had 5, totaling 346 scans. Following the initial CT scans, 84, 31, 30, 23, and 24 patients had additional CT scans on days 2–7, 8–14, 15–30, 31–60, and 61–90. The median time intervals between the first and subsequent CT scans were 5 (3), 10 (2), 25 (9), 47 (14), and 80 (12) days.

As shown in Table 1, the mean age of the total population was 47.5 ± 11.1 years, with 126 (81.8%) males. The prevalence rates of overweight, liver cirrhosis, ascites, HE, AKI, and infection were 29.2, 72.7, 78.6, 27.9, 9.1, and 89.0%, respectively. The median MELD-Na and AARC scores were 24.1 (6.6) and 9 (2), respectively. According to the EASL-CLIF-C diagnostic criteria, 65 patients (42.2%) were diagnosed with CLIF-C ACLF. During 90 days of follow-up, 106 (68.8%) patients survived without liver transplantation.

**Table 1:** Baseline characteristics of total cohort and high muscle vs. low muscle loss in patients with acute-on-chronic liver failure.

Variables	Total (*n* = 154)	High muscle loss (*n* = 77)	Low muscle loss (*n* = 77)	*p* value
Age (years)^a^	47.5 ± 11.1	48.2 ± 10.8	46.8 ± 11.3	0.446
Males, *n* (%)^c^	126 (81.8)	66 (85.7)	60 (88.2)	0.210
BMI (kg/m^2^)^b^	22.8 (4.3)	22.3 (4.7)	23.5 (4.2)	0.066
Overweight, *n* (%)^c^	45 (29.2)	19 (24.7)	26 (33.8)	0.215
Aetiology, *n* (%)^c^				0.226
Hepatitis B	91 (59.1)	49 (63.6)	42 (54.5)	
Alcohol	38 (24.7)	17 (22.1)	21 (27.3)	
Hepatitis B + Alcohol	11 (7.1)	7 (9.1)	4 (5.2)	
Other	14 (9.1)	4 (5.2)	10 (13.0)	
Liver cirrhosis, *n* (%)^c^	112 (72.7)	53 (68.8)	59 (76.6)	0.278
Ascites, *n* (%)^c^	121 (78.6)	62 (80.5)	59 (76.6)	0.556
HE, *n* (%)^c^	43 (27.9)	28 (36.4)	15 (19.5)	0.020
AKI, *n* (%)^c^	14 (9.1)	8 (10.4)	6 (7.8)	0.575
Infection, *n* (%)^c^	137 (89.0)	69 (89.6)	68 (88.3)	0.797
Total bilirubin (mg/dL)^b^	20.8 (15.4)	22.9 (16.8)	16.8 (12.9)	0.023
Albumin (g/L)^a^	29.3 ± 4.8	29.4 ± 5.0	29.3 ± 4.8	0.909
Creatinine (mg/dL)^b^	0.67 (0.24)	0.66 (0.30)	0.69 (0.23)	0.498
Sodium (mmol/L)^b^	137.2 (5.7)	137.1 (5.9)	137.3 (5.6)	0.715
Lactic acid (mmol/L)^b^	2.03 (0.95)	2.08 (0.89)	2.00 (1.00)	0.279
INR^b^	2.15 (0.72)	2.24 (0.74)	2.10 (0.68)	0.200
Hemoglobin (g/L)^a^	118.1 ± 23.2	117.5 ± 23.6	118.7 ± 23.0	0.753
WBC count (×10^9^/L)^b^	6.2 (4.6)	6.2 (5.0)	6.1 (4.3)	0.558
Platelet count (×10^9^/L)^b^	103.5 (82)	103 (79)	105 (88)	0.513
MELD-Na score^b^	24.1 (6.6)	25.2 (7.6)	22.6 (6.4)	0.010
AARC score^b^	9 (2)	9 (2)	9 (3)	0.015
CLIF-C ACLF, n (%)^c^	65 (42.2)	42 (54.5)	23 (29.9)	0.002
L3-SMI, cm^2^/m^2a^	46.0 ± 8.7	45.7 ± 8.7	46.3 ± 8.7	0.674
Sarcopenia, *n* (%)^c^	30 (19.5)	18 (23.4)	12 (15.6)	0.222
Change standardized to 30 days (cm^2^/m^2^)^b^	−2.71 (8.32)	−8.01 (10.3)	0.22 (5.00)	<0.001

Continuous variables are shown as mean ± standard deviation or median (interquartile range) for normally and non-normally distributed continuous variables, respectively. Categorical variables were presented as numbers (percentages). ^a^Independent sample t-test, bMann-Whitney U test, or cχ2 test was used to compare two groups of continuous or categorical variables.

### 3.2 Dynamic changes of L3-SMI

**Figure 2:**
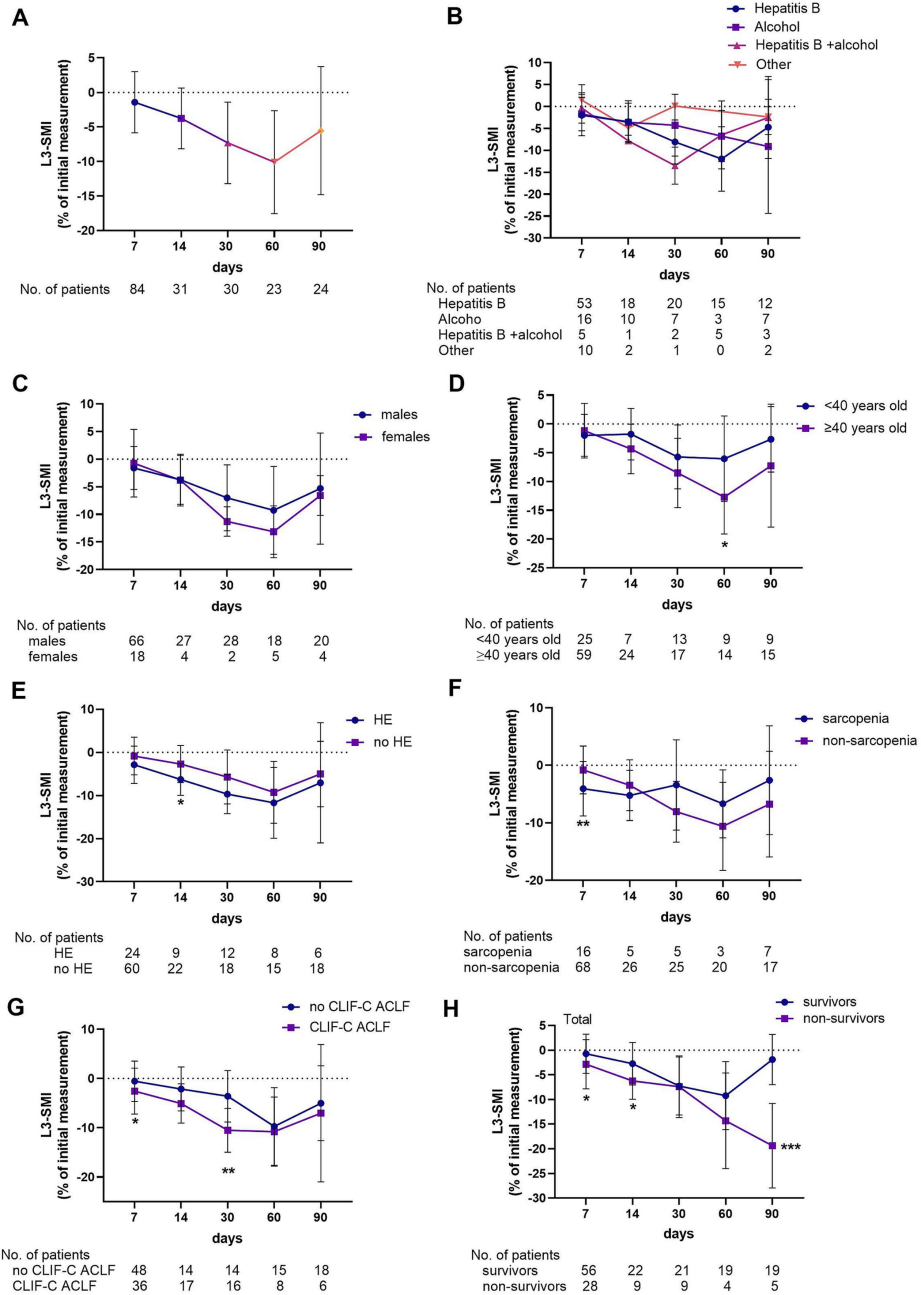
Percentage change rate of L3-SMI in the total study population **(A)** and grouped according to etiology **(B)**, gender **(C)**, age **(D)**, hepatic encephalopathy **(E)**, sarcopenia **(F)**, severity of the disease **(G)**, and 90-day prognosis **(H)**.

The mean L3-SMI of the total population was 46.0 ± 8.7 cm^2^/m^2^, and 30 (19.5) patients were categorized as having sarcopenia. The dynamic changes in muscle loss in ACLF were quantified, and an analysis was conducted to determine whether etiology, sex, age, HE, sarcopenia, and disease severity influenced the rate of muscle loss. The changes in L3-SMI were shown in Figure 2. In the total population, L3-SMI gradually declined over the course of the disease and began to recover within days 60–90. At 2–7, 8–14, 15–30, 31–60, and 61–90 days, the mean percentage decline was −0.83 ± 4.43, −3.76 ± 4.40, −7.30 ± 5.89, −10.10 ± 7.45, and − 5.53 ± 9.26, respectively (Figure 2A).

Considering the influence of demographic indicators on muscle mass changes, the study population was further grouped according to etiology, sex, and age. When grouped by etiology, no difference was observed in the percentage change in L3-SMI at each time point among patients with hepatitis, alcoholic liver disease (ALD), hepatitis combined with ALD, or other causes (Figure 2B). Similarly, no difference was observed in the change in L3-SMI between males and females at any time point (Figure 2C). However, significant reductions in L3-SMI were noted in patients over 40 years old compared with those under 40 at 31–60 days (−12.7 ± 6.43 vs. -6.05 ± 7.44, *p* = 0.033). Although a decreasing trend was observed in other periods, the difference was not statistically significant (Figure 2D).

The study population was grouped according to the presence of HE and sarcopenia, and disease severity. Patients with HE had a higher rate of decrease in L3-SMI than those without HE on days 8–14 (−6.30 ± 3.66 vs. -2.72 ± 4.31, *p* = 0.037; Figure 2E). Similarly, patients with sarcopenia had a higher rate of decrease in L3-SMI than those without sarcopenia on days 2–7 (−4.08 ± 4.73 vs. -0.80 ± 4.15, *p* = 0.007; Figure 2F). The percentage decline in L3-SMI in patients with CLIF-C ACLF in the early stage of the disease (2–7, 8–14, and 15–30 days) was higher than that in patients with no CLIF-C ACLF ((−2.56 ± 4.65 vs. -0.57 ± 4.10, *p* = 0.04), (−5.09 ± 3.99 vs. -2.14 ± 4.46, *p* = 0.062), and (−10.53 ± 4.44 vs. -3.61 ± 5.23, *p* = 0.001), respectively; Figure 2G). Finally, according to the 90-day prognosis, the L3-SMI of both survivors and non-survivors decreased over time in the first 2 months. However, that of the survivors began to improve in the third month, while that of the non-survivors further decreased, with a statistically significant difference between the two groups at 61–90 days (−1.89 ± 5.09 vs. -19.38 ± 8.58, *p* < 0.001; Figure 2H).

### 3.3 The association of nutrition indicators and skeletal muscle wasting

**Figure 3:**
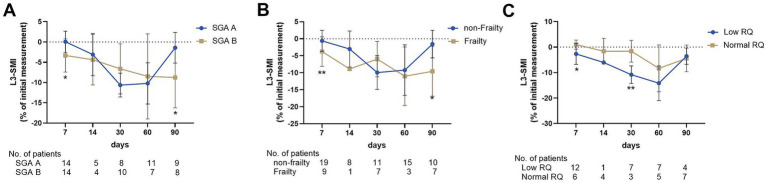
Percentage change rate of L3-SMI grouped by SGA score **(A)**, frailty **(B)**, and RQ **(C)**.

Of the total population, 59 patients who underwent nutritional assessment at admission were retrospectively analyzed from a prospective cohort from August 2021 to August 2022. The relationship between baseline nutritional status and skeletal muscle wasting in patients with ACLF was further analyzed. Among the 59 patients, 32 (54.2%) were diagnosed with malnutrition based on SGA scores. Moreover, the patients had a median liver frailty index of 3.74 (0.9), and 17 patients (28.8%) were diagnosed with frailty. The decline in L3-SMI in the early course of ACLF (2–7 days) was greater in patients with malnutrition diagnosed by SGA score and frailty defined by high LFI than in those who were well-nourished (−3.32 ± 4.10 vs. 0.05 ± 2.63, *p* = 0.017; Figure 3A) and non-frail (−5.51 ± 2.91 vs. -0.35 ± 3.13, *p* < 0.001; Figure 3B). Among the 59 patients in the prospective cohort, 32 underwent a nutrition metabolism test, with a median RQ of 0.82, and 18 (56.3) were categorized as having a low RQ (< 0.80). Interestingly, the decline in L3-SMI in patients with lower RQ in 2–7 (−2.67 ± 4.13 vs. 0.99 ± 1.78, *p* = 0.019) and 15–30 days (−10.82 ± 3.46 vs. 1.62 ± 4.27, *p* = 0.007) was greater than that in patients with a normal RQ (Figure 3C).

### 3.4 The effect of skeletal muscle loss on 90-day mortality

We next investigated whether the degree of muscle loss influenced 90-day mortatliy in ACLF patients. The median interval between the initial and final CT scans was 8 days (IQR: 5–27 days). Due to the absence of standardized CT protocols with fixed time intervals in ACLF patients, we calculated the absolute change in muscle mass normalized to a 30-day period. The median absolute change in L3-SMI, standardized to 30 days, was −2.71 (8.32) cm^2^/m^2^. Using this median value as a threshold, patients were categorized into high and low muscle loss groups. The high muscle loss group exhibited a median decrease of −8.01 (10.3) cm^2^/m^2^, whereas the low muscle loss group showed a median change of 0.22 (5.00) cm^2^/m^2^.

**Figure 4:**
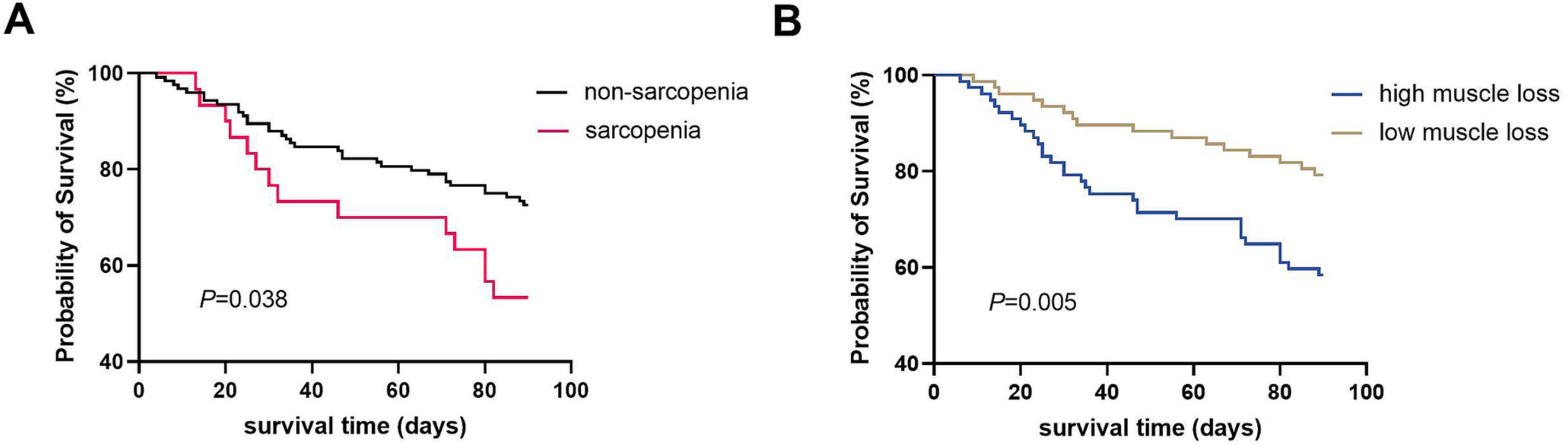
Kaplan–Meier curves of 90-day survival probability in patients with ACLF according to pre-existing sarcopenia **(A)** and the dynamic change of muscle mass during the course of ACLF **(B)**.

The baseline characteristics of the high and low muscle loss groups were shown in Table 1. The two groups had comparable ages, BMI, and incidence of complications. However, the MELD-Na [25.2 (7.6) vs. 22.6 (6.4), *p* = 0.010] and AARC [9 (2) vs. 9 (3), *p* = 0.015] scores and the presence of CLIF-C ACLF (54.5% vs. 29.9%, *p* = 0.002) were significantly higher in the high muscle loss group than in the low muscle loss group. Patients with sarcopenia at admission exhibited a significantly lower 90-day survival probability compared to those without sarcopenia (53.3% vs. 72.6%, *p* = 0.038; Figure 4A). Similarly, the 90-day survival probability was significantly reduced in patients with high muscle loss relative to those with low muscle loss (58.4% vs. 79.2%, *p* = 0.005; Figure 4B). Univariate and multivariate Cox regression analyses were used to further analyze independent risk factors for 90-day mortality. Age [hazard ratio (HR) = 1.038; 95% CI = 1.009–1.069, *p* = 0.011] and high muscle loss (HR = 2.059; 95% CI = 1.122–3.780, *p* = 0.020) were independently associated with 90-day mortality in multivariate regression analysis (Table 2).

**Figure 5:**
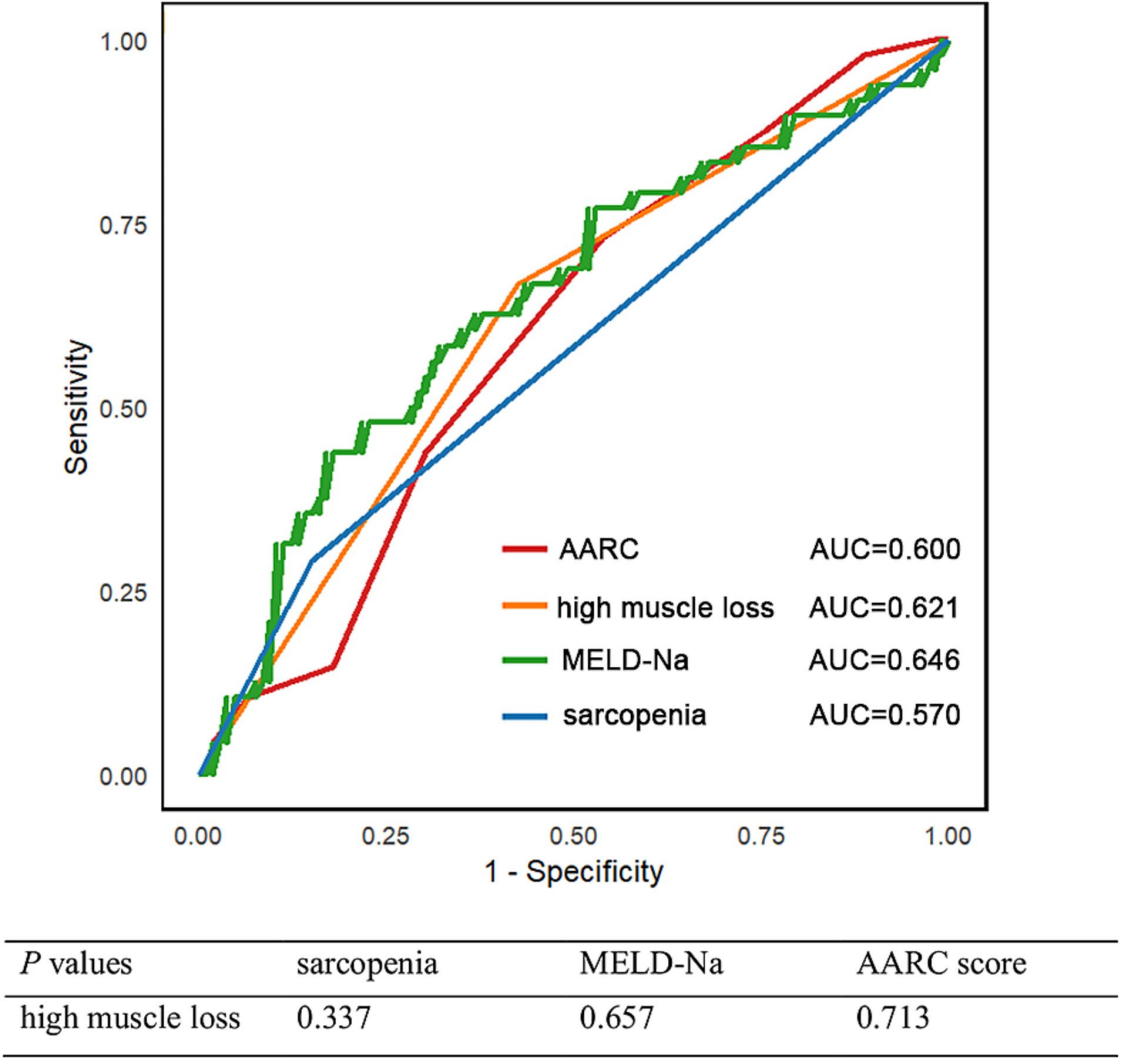
Time-dependent ROC curve for 90-day mortality and corresponding AUC values of sarcopenia, low muscle mass, and disease severity indicators.

Although baseline sarcopenia did not emerge as an independent risk factor for 90-day mortality in ACLF patients when analyzed alongside key covariates such as high muscle loss, MELD-Na and AARC score, the existing body of literature sonsistently underscored its prognostic significance in ACLF outcomes. Given this established clinical relevance, we conducted a comparative analysis of predictive performance among sarcopenia, low muscle mass, and disease severity indicators for 90-day mortality using ROC curve analysis. The evaluation revealed that high muscle loss demonstrated superior predictive capability (AUC = 0.621) compared to baseline sarcopenia (AUC = 0.570), although this difference did not reach statistical significance. These findings suggest that dynamic assessment of muscle loss may offer enhanced prognostic value over static baseline sarcopenia measurements in predicting 90-day mortality (Figure 5).

**Table 2:** Univariate and multivariate Cox regression analysis for 90-day mortality in patients with acute-on-chronic liver failure.

Variables	Univariate		Multivariate	
	HR (95% CI)	*p* value	HR (95% CI)	*p* value
Age (years)^a^	1.045 (1.017–1.073)	0.001	1.038 (1.009–1.069)	0.011
Obesity, *n* (%)^c^	0.601 (0.299–1.205)	0.152		
Liver cirrhosis, *n* (%)^c^	1.218 (0.621–2.387)	0.566		
Ascites, *n* (%)^c^	3.296 (1.184–9.177)	0.022	2.257 (0.790–6.449)	0.128
HE, *n* (%)^c^	2.094 (1.179–3.719)	0.012		
AKI, *n* (%)^c^	1.555 (0.661–3.658)	0.312		
Infection, *n* (%)^c^	1.508 (0.542–4.197)	0.432		
Albumin (g/L)^a^	0.973 (0.919–1.029)	0.338		
Hemoglobin (g/L)^a^	0.992 (0.980–1.004)	0.179		
WBC count (×10^9^/L)^b^	1.023 (0.965–1.085)	0.437		
Platelet count (×10^9^/L)^b^	0.998 (0.993–1.003)	0.487		
MELD-Na score^a^	1.043 (1.008–1.080)	0.016	1.019 (0.972–1.067)	0.440
AARC score^b^	1.174 (1.006–1.371)	0.042	1.068 (0.866–1.318)	0.538
Sarcopenia, *n* (%)^c^	1.904 (1.021–3.550)	0.043	1.430 (0.724–2.826)	0.303
High muscle loss	2.809 (1.485–5.312)	0.001	2.059 (1.122–3.780)	0.020

Age, ascites, MELD-Na score, AARC score, sarcopenia, and high muscle loss were included in multivariate analysis.

## 4 Discussion

Through a retrospective analysis of abdominal CT images (including the L3 level) of 154 patients with ACLF at different time points, this is the first study to investigate the dynamic changes in skeletal muscle mass and poor prognosis. In the first 2 months of ACLF progression, muscle mass gradually decreased. Survivors started to recover in the third month, whereas non-survivors continued to experience a decline. The muscle loss rate is related to age, HE, sarcopenia, disease severity, and nutritional status. Furthermore, dynamic skeletal muscle mass depletion, rather than sarcopenia at baseline, was an independent risk factor in the 90-day period.

Sarcopenia is prevalent in cancer and chronic diseases and is closely related to adverse clinical outcomes such as increased mortality and morbidity (18). However, most studies have focused on evaluating sarcopenia at a specific time, and few have explored dynamic changes in skeletal muscle mass. It is well known that skeletal muscle mass gradually decreases with age. A 12-year cohort study showed that muscle mass decreases by approximately 1.4% yearly due to physiological aging (19). Unlike age-related muscle mass loss, the rate of decline in muscle mass caused by a disease is faster and differs among different diseases. Hanai et al. retrospectively analyzed 149 patients with liver cirrhosis and reported that the median change in L3-SMA per year in all patients was −2.2% (20). In patients with HCC, the skeletal muscle mass decreased more significantly. Kobayashi et al. retrospectively evaluated 102 patients with HCC treated with transcatheter intra-arterial therapies and found that the L3-SMI of patients with HCC decreased by an average of 3.5% within 6 months (21). In contrast to chronic diseases, skeletal muscle mass decreases faster in patients with severe acute diseases. This retrospective study included 95 consecutive patients hospitalized for COVID-19. The cross-sectional areas of the pectoral muscles (PM) and erector spinae muscles (ESM) were measured using chest CT, and the time was standardized for 30 days to assess muscle changes at 30-day intervals. An average decrease of 2.64% in PM and 1.86% in ESM has been observed (10). Cox et al. measured L3-SMI in 47 patients with sepsis using CT at admission and at 3 and 12 months and found that L3-SMI at 3 months was 8% lower than that at baseline (22). In this study, given that skeletal muscle mass did not decline consistently with the progression of ACLF, the percentage change was analyzed based on distinct time intervals. In the overall analysis, the rates of muscle mass change in patients with ACLF at 2–7, 8–14, 15–30, 31–60, and 61–90 days were − 0.83 ± 4.43%, −3.76 ± 4.40%, −7.30 ± 5.89%, −10.10 ± 7.45%, and − 5.53 ± 9.26%, respectively. The rate of muscle mass loss in patients with ACLF was significantly higher than that in patients with liver cirrhosis and cancer.

The underlying etiology of liver disease can affect skeletal muscle mass by altering muscle metabolism. Welch et al. included 83 patients with cirrhosis (nonalcoholic fatty liver disease, *n* = 26; ALD, *n* = 39; viral hepatitis, *n* = 18) and found that the mean percentage change in the psoas area in patients with liver cirrhosis was −3.52 ± 0.45 per 100 days. Among patients with liver cirrhosis, those with ALD had the lowest initial muscle area and the most rapid rate of muscle loss (23). In this study, there was no difference in the rate of muscle loss among the different etiologies at any time point. The potential reason for our results is that skeletal muscle metabolism was not affected by the etiology in a short time. Previous studies have defined a decline in muscle mass after 40 years in healthy populations and patients with ACLF (3, 16). We further investigated whether the decline rate of muscle mass in patients with ACLF at different ages (< 40 years and ≥ 40 years) was different. It was found that the decline rate of patients aged ≥40 years had a higher trend than that of patients aged <40 years during the course of the disease, which may imply that patients who are older adults are more likely to have negative nitrogen balance and nutritional risk. Disease severity can significantly affect muscle mass loss in patients with ACLF. CLIF-C ACLF was diagnosed based on the number of organ failures, which were more severe in patients with the condition than in those without it. This study found that the rate of skeletal muscle mass decline in patients with CLIF-C ACLF was significantly higher than that in patients without the condition in the first month. Similarly, Puthucheary et al. evaluated muscle loss through serial ultrasound measurements of the femoral cross-sectional area in patients in the intensive care unit on days 1, 3, 7, and 10. The study found that muscle loss occurred early and rapidly in the first week of critical illness, and the loss was more severe among patients with multi-organ failure than among those with single-organ failure (8).

ACLF is often accompanied by severe gastrointestinal symptoms and absorption disorders, resulting in an insufficient intake of energy and proteins. The incidence of malnutrition is high in patients with ACLF. Malnutrition may lead to adverse physical effects that commonly manifest as a loss of muscle mass (9). Frailty is characterized by decreased physiological reserves and increased vulnerability to stressors. A previous study reported that L3-SMI (*β* = −0.260, *p* < 0.001) was an independent risk factor for frailty (24). Simultaneously, frailty may also contribute to loss of muscle mass through inactivity. It has been reported that muscle mass decreases by 5.5% ± 0.6% after 7 days of muscle disuse (25). In this study, we observed a more severe loss of muscle mass in patients with malnutrition and frailty during the early course of ACLF. The systemic inflammatory response is a hallmark of ACLF and is energy-consuming. Nutrients (glucose, amino acids, and lipids) are reallocated for synthesizing cytokines and acute-phase proteins (26). RQ is the ratio of carbon dioxide production to oxygen consumption (CO_2_/O_2_), reflecting substrate metabolism. A low RQ indicates that glycogen utilization is impaired, and lipid metabolism and protein catabolism are enhanced as energy sources (27). In this study, patients with low RQ had a substantial loss of muscle mass during the early course of ACLF, indicating greater muscle loss due to increased protein catabolism. Further studies are required to investigate interventions to improve nutrition, frailty, and nitrogen reserves to prevent muscle loss.

Other pathophysiological perturbations also account for pronounced muscle wasting in the context of ACLF. For instance, these patients are inclined to acute deterioration and dysregulated homeostasis internally pertinent to inflammatory milieu. Persisent TNF-*α* may trigger mitochondria swelling and membranous alterations, resulting in subsequent deficiency toward energy supply and muscular protein synthesis (28). Moreover, hyperammonemia, a common characteristic of this aggravating condition, can instigate autophagic process, oxidative stress and proteolysis in the skeletal muscle cells (29). The bursting of myostatin upon myotoxic ammonia accumulation also curtail muscular protein turover (30).

Sarcopenia is prevalent in chronic liver disease, and acute exacerbation of muscle loss may occur in ACLF. Notably, a greater magnitude of muscle mass loss is an important prognostic factor. When the absolute change was standardized to 30 days, dynamic loss of muscle mass, rather than lower baseline muscle mass, was an independent risk factor for 90-day mortality. However, the MELD-Na and AARC scores were not significant in multivariate Cox regression analysis, indicating that a dynamic assessment of the clinical course of patients with ACLF is essential. No unified definition exists for high muscle loss. According to a previous method, we divided the patients into high and low muscle loss groups based on the median value. The adverse prognostic effects of the magnitude of muscle loss were analyzed and laid the foundation for an in-depth study. These findings underscore the critical importance of monitoring dynamic changes in muscle mass in patients with ACLF. Clinically, this highlights the need for early identification and targeted interventions in high-risk subgroups, particularly older patients and those with HE, malnutrition, sarcopenia, or critical illness. To mitigate further muscle loss, a multidisciplinary approach should be adopted, including regular nutritional assessments, personalized dietary plans, and timely supplementation with essential nutrients. Additionally, integrating muscle mass evaluation into routine clinical practice—using accessible tools such as CT-based measurements—can help stratify patients and guide therapeutic decisions. These measures may not only improve muscle preservation but also potentially enhance overall clinical outcomes in ACLF patients.

This study has a few limitations. First, this retrospective study only included patients who underwent two or more CT scans within 90 days of admission, which may have caused selection bias. Second, only a few patients underwent SGA, frailty evaluation, and energy metabolism tests, and we only investigated the impact of baseline malnutrition, frailty, and low RQ on muscle mass loss in the ACLF phase. Additionally, because of the retrospective design, it was impossible to determine whether patients received sufficient nutritional supplementation during the course of ACLF. Third, we speculated that inflammation and catabolism in ACLF are detrimental to muscle mass loss; however, no related biomarkers were measured. Further studies are warranted to investigate the underlying mechanisms and possible therapies for muscle loss over the course of ACLF. Finally, this was a single-center study with a relatively small sample size, which may limit its generalizability to other patient populations. Moreover, the lack of external validation in an independent cohort restricts the broader applicalibily of our findings. Future studies should include external validation to confirm the robustness and generalizability of our results. Nonetheless, our study lays the foundation for conducting multicenter prospective studies.

In conclusion, we demonstrated that a significant reduction occurs in skeletal muscle mass during the course of ACLF and that the degree of reduction is associated with disease severity and nutritional status. Furthermore, dynamic changes in muscle loss, rather than low muscle mass at baseline, were independent risk factors for 90-day mortality. This study underscores the significance of evaluating dynamic muscle loss and the need to reverse muscle loss when managing patients with ACLF.

## Data Availability

The raw data supporting the conclusions of this article will be made available by the authors, without undue reservation.
